# Evaluation of a charge nurse leadership development program

**DOI:** 10.1097/nmg.0000000000000032

**Published:** 2023-07-03

**Authors:** Kelly Medero, Jama Goers, Mary Beth Flynn Makic

**Affiliations:** At Denver Health in Denver, Colo., **Kelly Medero** is the director of critical care, and **Jama Goers** is the director of nursing education, research, and innovation. **Mary Beth Flynn Makic** is a professor at the University of Colorado College of Nursing, Anschutz Medical Campus in Aurora, Colo.

## Abstract

A pilot study evaluated the change in charge nurses' perception of their leadership skills after engaging in a 4-month structured leadership program. Based on a self-assessment, multimodal education using authentic leadership tenets and an appreciative inquiry framework increased participants' confidence in their skills.

**Figure:**
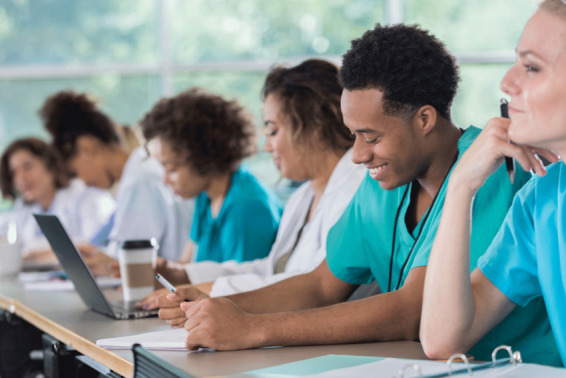
No caption available.

The hospital-based charge nurse (CN) can make or break the team dynamic on the shift. They have extensive responsibilities, but it's more than what they do that's critical, it's how they do it. CNs must use effective communication, influence, delegation, interpersonal relationship-building, and vision-setting skills while executing essential tasks. This is crucial because leadership skills have become more important than ever to reduce nursing turnover postpandemic.^1^ Unfortunately, CNs often feel unprepared to effectively assume these essential leadership roles.^2^-^4^

The nursing leadership at a large, urban teaching hospital in Colorado recognized the value of the CN role. The hospital offered a CN orientation class. Beyond CN orientation, there were organizational development classes, but the content wasn't specifically taught to the role of the CN and participation was lacking. Because of the lack of continuous education focused on developing CN leadership skills within the organization, nurse leaders believed that creating ongoing CN-specific education could be beneficial. Nurse leaders proposed a CNO-approved evidence-based pilot program to support the strategic priority of investing in staff workforce development opportunities. The primary purpose of this pilot study was to evaluate CN participants' self-perception of improved confidence in leadership skills after engaging in a 4-month structured leadership program.

## What does the literature say?

The literature suggests that leadership development programs are beneficial. Kramer and Davies conducted a review of nine studies and concluded that formal orientation and ongoing leadership support is required for CNs to be successful.^5^ Krugman and colleagues found a significantly higher rating of CNs inspiring a shared vision for direct care nurses after attending an educational program.^6^ Nurse leader education was found to improve patient safety culture and outcomes.^7^,^8^ Dowling Dols and colleagues linked increased patient satisfaction scores regarding nurse communication to a CN training program.^3^ Finally, multiple studies showed that CNs have a more positive perception of their job performance after training in leadership skills.^2^-^4^

Although the benefit of ongoing leadership development has been defined, there are no standardized competencies for CNs.^4^,^5^,^8^,^9^ Training topic themes found in the literature focus on communication, developing relationships, promoting teamwork, and understanding one's leadership style and self-awareness.^2^-^5^,^7^,^8^,^10^,^11^ Some courses included training on emotional intelligence.^4^ One theme that was absent in the reviewed literature was training in diversity, equity, inclusion, racism, and bias.

## Theoretical framework

It was important to design this program with theories that CNs could apply to day-to-day work and, in turn, use when leading improvement work on their clinical units. The project leader selected Patricia Benner's From Novice to Expert theoretical framework; although often applied to clinical skills, this theory also has relevance to nursing leadership.^12^,^13^ Both a novice and experienced CN may be capable of managing a leadership situation, but the outcome of how well it's managed is attributed to the individual's expertise, aligning with this framework.

The program also used Appreciative Inquiry (AI) as the model for change management.^14^ The core of AI follows the key theme that improvement work in an organization is a mystery to be embraced as opposed to a problem to be solved.^14^ This idea was helpful to make change management approachable within the context of the CN role.

## Workshop series

The Charge Nurse Leadership Development Workshop Series (CNLDWS) was created to evaluate if implementation of a 4-month structured leadership development program increased CNs' self-perception of confidence in their leadership skills. The project leader also explored commonalities in factors that motivated participant involvement in leadership development. Participants were included if they worked in a hospital-based CN position regardless of years in the role, were in good employment standing, and were willing to attend the classes. There were 111 eligible CNs at the time of course offering; however, because this was a pilot program, the recruitment was limited to 12 CNs from diverse units. To recruit participants, the project leader sent targeted emails to eligible staff and advertised at daily CN patient staffing meetings. Participation was voluntary, without penalty for course discontinuation, and continuing-education credits were provided.

The CNLDWS was a 4-month course that consisted of four 90-minute, in-person classes (one per month) followed by an email with related optional multimodal education, such as podcasts, articles, and blogs. CNs were compensated for attendance. All classes were taught by an experienced nurse leader within the organization who filled the role of course lead. The classes were held in four consecutive months (May through August).

The CNLDWS curriculum was thoughtfully designed to be specific to the CN role, based on the evidence and the organization's leadership performance expectations. The courses were structured to build on the knowledge learned from class to class and enriched through group discussion. Course topics with subtopics are outlined in Table 1. The first hour of each class provided didactic education. Classes two through four included discussions about applying learned knowledge. The final segment of each class was dedicated to a breakout group AI exercise.

**Table 1:** Course content overview

	Class 1	Class 2	Class 3	Class 4
**Topic**	Understanding others	Leadership	Communication in charge	Influencing others: Techniques to motivate
**Subtopics**	Diversity (generational, racial) Equity Inclusion Conscious and unconscious bias Hidden rules	LPI interpretation LPI: Practices of exemplary leadership Authentic leadership	Critical conversations How to prepare for and execute Giving and receiving feedback Documenting conversations Critical conversations: Patients/families	Influence: Why it matters Ways to influence Picking the right tactics Recognizing when others are influencing you
**AI exercise**				
**Discovery**	Within your role as a charge nurse, what actions do you take that support an inclusive work and care environment? On your unit, what practices, processes, or cultural norms do you have that support an inclusive work and care environment?	Describe one of your best shifts as a charge nurse. Was there a challenge or a problem? Discuss the situation and what you/others did to make the situation successful. On your poster, write skills, behaviors, or actions that made you successful that day.	What makes you a great communicator? On your poster, write skills, behaviors, or actions that made you successful.	What factors or tactics do you rely on most when influencing? When have you been successful influencing?
**Dream: In a perfect world...**	Within your role as a charge nurse, what actions would you take to support an inclusive work and care environment? What practices, processes, or cultural norms would your unit have that would support an inclusive work and care environment?	What skills, behaviors, or actions would describe the best charge nurse you could be? What would “better” look like to build on what you already do well? Think of the best and worst peers, leaders, and people outside of work who have influenced you. What did they do that you want to replicate or avoid?	I would be more confident in my crucial conversation skills if I could ______ better. Listen? Respond in the moment? Pick the right words? De-escalate? Maintain positive body language? Better plan for the discussion? Provide better follow-up after? Give more positive feedback? Address issues in the moment? Actually have the conversation?	How would you describe a top-notch influencer? Think of a colleague, peer, or boss who's a great influencer. What do they do that seems to work?
**Design**	Within your role as a charge nurse, what actions will you start to support an inclusive work and care environment? What practices, processes, or cultural norms from the dream should your unit start that would support an inclusive work and care environment?	From the list of the dream state, what are your top one to three leadership skills, actions, and behaviors that you'll start today? Are there any barriers on your unit or team that may hold you back from performing these actions or behaviors? What could you do to break down these barriers?	What could you do to get to your dream state?	What are one to two actions you could start today to be a better influencer?
**Destiny**	How will you know if you've made the personal change you've committed to? What steps will you take to bring your unit's ideas back to your leadership team?	How will you know if you've made the personal change you've committed to?	How will you know if you've made the personal change you've committed to?	How will you know if you've made the personal change you've committed to?

After each class session, the course leader distributed an email to participants with a brief course evaluation and optional self-driven learning. There were also six weekly emails sent after the fourth class to foster ongoing engagement in self-paced learning. Multimodal self-study options from nursing and nonnursing sources included journaling; free online podcasts and videos; self-assessment tools; and publications such as books, journal articles, website blogs, and professional documents. Participants had the option be compensated up to 2 hours per week for completing this work remotely with manager approval, as supported by organizational policy.

Confidence in leadership skills was measured through a pre-post assessment using the *Leadership Practices Inventory (LPI): Self*.^15^ Permission to use the tool was obtained from John Wiley & Sons, Inc. The self-assessment tool is based on Kouzes and Posner's transformational leadership model, using the Five Practices of Exemplary Leadership as a foundation: *Model the Way, Inspire a Shared Vision, Challenge the Process, Enable Others to Act,* and *Encourage the Heart*.^16^ There are 30 questions that prompt respondents to rate how frequently they use each leadership behavior on a 10-point Likert-type scale. Posner published a review of the reliability and validity of the tool based on studies that used the LPI and a normative database.^17^ CN participants created an anonymous identification code that could be used to pair responses in self-assessment before and after completing the course. Factors that motivated program participation were assessed through a three-question qualitative survey.

This program evaluation was reviewed and approved as nonhuman subject research by the University of Colorado College of Nursing Bridge Committee and the hospital's Quality Improvement Committee with the authority of the Colorado Multiple Institute Review Board at the University of Colorado-Denver.

## Enriched leadership behaviors

There were 12 CN participants who were predominately White (67%) and female (100%), and 58% were 30 to 49 years old (see Table 2). The average range of registered nursing practice was 11 to 15 years, and 92% had less than 10 years of experience as a CN (Table 2). Twelve CNs engaged in the program evaluation project and completed the preintervention LPI, 11 CNs completed the postintervention survey, and 9 surveys were paired for analysis. Unpaired responses were included in the independent *t* test analysis.

**Table 2:** Participant demographics (N = 12)

Characteristics	Participants %	n
Age		
20-29	16.67	2
30-39	33.33	4
40-49	25	3
50-59	25	3
Identifying Gender		
Female	100	12
Race		
White or Caucasian	66.67	8
Hispanic or Latin	25	3
Prefer not to answer	8.33	1
Years of RN experience		
1-5	16.67	2
6-10	16.67	2
11-15	41.67	5
16-20	16.67	2
21-25	0	0
26-30	8.33	1
Years on current work unit in any nursing role (certified nursing assistant, direct care RN, charge RN, other)		
<1	8.33	1
1-5	25	3
6-10	16.67	2
11-15	33.33	4
16-20	8.33	1
21-25	8.33	1
Years in permanent charge RN position		
<1	33.33	4
1-5	33.33	4
6-10	25	3
11-15	0	0
16-20	0	0
21-25	8.33	1
Primary work shift (more than 50% of time)		
Days	41.67	5
Nights	50	6
Rotate days and nights	8.33	1

The primary analysis (n = 9 paired surveys) found participants reported an increase in self-assessed leadership behaviors with preintervention LPI scores (*M* = 210.8, 95% CI [190.5, 231.2]) to postintervention *(M* = 238.5, 95% CI [216.0, 261.1]) with a mean difference of 23.7 (95% CI [10.1, 37.2]), *t* = 4.03, *P* = .004). An independent *t* test (N = 12) showed similar results, with a mean difference of 32.3 (95% CI [6.5, 58.2], *t* = 2.6, *P* = .017). Analysis of the Five Practices of Exemplary Leadership found a statistically significant increase in all domains (see Table 3).

**Table 3:** Comparison of pre- and post-CNLDWS CN LPI scores

Paired *t* test Leadership Practices Inventory (LPI) scores by five exemplary practices and total score												
Charge nurse scores		Before CNLDWS		After CNLDWS								
	Mean	SD	Mean	SD	n	Differences	95% CI for mean differences			*t*	*df*	*P* ∗
Five exemplary practices												
Model the Way	45.889	6.698	48.556	5.747	9	2.667	0.393	to	4.94	2.704	8	0.027∗
Inspire a Shared Vision	36.000	9.151	42.222	6.629	9	6.222	0.296	to	12.149	2.421	8	0.042∗
Challenge the Process	40.111	8.343	45.444	7.650	9	5.333	3.060	to	7.607	5.409	8	0.001∗
Enable Others to Act	48.778	3.930	52.889	3.655	9	4.111	1.381	to	6.841	3.473	8	0.008∗
Encourage the Heart	44.111	7.061	49.444	7.264	9	5.333	1.832	to	8.835	3.512	8	0.008∗
Total LPI score	210.833	30.759	238.556	29.305	9	23.667	10.127	to	37.206	4.031	8	0.004∗

Abbreviations: CI = confidence interval; CNLDWS = Charge Nurse Leadership Development Workshop Series; *df* = degrees of freedom; *P* = probability; SD = standard deviation

∗ = significant

Qualitative survey responses revealed that CNs shared similar comments for why they participated in the program. Responses showed a desire for growth in leadership skills for personal improvement and to be better leaders on their units. One participant wrote: “To help in setting up an excellent unit culture, a successful and supported team, and to promote excellent practices on our unit.” The postintervention inquiry about how the program helped CNs on their leadership journey had an overwhelmingly positive response, revealing perceived value of the content and appreciation of the multimodal educational options. Eight participants completed a 45-day postclass survey, with all positively responding that they implemented one thing they learned into practice.

After initiation of the course, one unexpected benefit was that some participants requested discussions about career progression, critical conversation planning consultation, and requests for ongoing mentorship from the course leader. The nature of these discussions was very positive and, despite working relationships in some cases, had not occurred prior to the course.

## Implications for practice

The results of this evidence-based leadership skill development program support and expand on current literature. Although the small sample size is a limitation, the positive findings support the work of others.^5^-^8^ Krugman and colleagues used the LPI in their longitudinal CN leadership study over the 16-year period, ultimately concluding that CN leadership development contributed to a positive workplace culture.^6^ LPI scores weren't reported to be paired within the Krugman study; however, exploring paired participant self-evaluation of leadership skill growth builds on that work.

The LPI has been used to assess the leadership skills of CNOs in two studies.^18^,^19^ The results of this CN program evaluation were similar in how participants rated their leadership behaviors within the Five Practices of Exemplary Leadership.^15^ The domain *Enable Others to Act* had the highest mean self-assessment score postprogram.^18^,^19^ This demonstrates that nurses with varying degrees of leadership scopes value this domain's key behaviors of fostering collaboration and strengthening others.

There was value to the CNLDWS beyond self-assessment using the LPI. Participants reported that learning application of the principles of authentic leadership, improved communication skills, and diversity training were the most helpful. Homer and Ryan also found that communication was valued by CNs as a top skill set to be included in education.^4^ Diversity training (racial diversity, generational diversity, equity, inclusion, and unconscious bias) topics covered within the CNLDWS, although not specifically called out in the literature, were considered important for inclusion in CN educational classes. During session discussions, participants self-reported that they'd either personally experienced or witnessed an event associated with racist comments made by patients or visitors. The group shared how they handled the situation and what techniques could be used during a future incident.

When designing the course, investigators didn't yet appreciate how often negative interactions associated with racism were occurring. After the conclusion of the CNLDWS, the American Organization for Nursing Leadership (AONL) published their Core Competencies for Nurse Leadership to guide the practice of all nursing leadership roles.^20^ This new framework addressed diversity, equity, and inclusion topics with more specificity than previous publications. The content taught in the CNLDWS addressed the actions listed in the framework. Increasing diversity training to frontline leaders is essential when addressing the dynamically changing needs in practice environments.^21^-^23^

An impactful taught topic that emerged during group discussion was about “hidden rules,” which are unspoken social cues within a group that are expected to be followed.^24^ Some participants began the discussion stating their unit didn't have hidden rules but quickly changed their opinion and listed examples. One participant shared that the nurses' station had an unmarked seat that the CNs always sat in to lead the team shift huddle. Due to its location, new staff members would often sit in this chair on their first day, only to be asked to get out of the chair when the huddle started. This hidden rule sets the new employee up for embarrassment and failure.

Examples shared by the participants led them to acknowledge that nursing units aren't always inclusive environments. This inspired further discussion regarding tactics for how the CNs could influence a culture of inclusivity, such as those proposed by Schmidt and colleagues in their Code of Conduct for Inclusion and Diversity.^25^ This session seemed to be especially impactful considering many teams were hiring new staff.

Participants found value in the multimodal self-study education to address generational preferences for teaching and learning. Evidence suggests modalities such as discussion boards are preferred by baby boomers and Generation X; however, Generations Y and Z prefer more digital sources with visually engaging platforms.^26^,^27^ The postclass learning also allowed participants to be compensated to engage with the content that interested them without being penalized if they chose not to.

There were 10-18 educational opportunities sent after each class. Participants discussed what they learned from the multimodal education at the beginning of each class, which revealed evidence that learners engaged with each type of education offered. There didn't appear to be one preferred modality within the cohort. Multimodal education provided participants the flexibility to engage with the material in the method, time, and location that was convenient to their busy lifestyles.

The in-person classes were essential because they allowed for robust discussion and peer-led learning. Participants occasionally missed class due to scheduling conflicts or oversights. It was helpful to assess expected attendance for each class and reschedule when needed. Text message reminders for upcoming classes encouraged attendance.

Finally, in alignment with Delamater and Hall's summation, it matters that an experienced clinical nurse leader taught the course and could provide real-life application of content to the CN role.^2^ This was important because class discussion often circled back to how to coach staff members on the teams. Additionally, because the CN is both the staff advocate and the liaison to the nurse manager, they must be skilled at influencing their supervisor. It was helpful that the class leader could explain the nurse manager's perspective and offer tactics to help the CN increase the likelihood that they'll be heard.

Program strengths were that CNs with varying years of experience found value in the content and reported that they applied what they learned. This pilot program provided paid time for CNs who participated as a strategy to support professional development. Participants were compensated for attending the 90-minute sessions and were provided education time for self-paced learning. By offering paid time to learn, the organization demonstrated their commitment to professional growth by encouraging nurses to become more engaged in their professional development.

Efforts to continue the program have been through budgetary processes at the organization to further develop and sustain this pilot program in support of CN leadership skill attainment and growth. However, there are some limitations to the program. A larger sample size of CN engagement may have provided a deeper understanding of the impact of the CNLDWS on the CNs' perceptions of their skills. The evaluation was limited to the participant's self-perception and didn't include a 360-degree assessment or unit-based outcomes. Finally, although it was informed by best evidence to teach leadership skills to CNs, the program evaluation was influenced by the organization's culture and isn't generalizable.

## A valuable investment

The CNLDWS provided high-value support to CNs with diverse levels of experience. Ideally, growing CN self-perceived leadership strengths will also benefit the organization, unit, and patient populations served. The program was informed by and tied to professional nursing recommendations, the works of thought leaders, and the organization's expectations of CNs' daily work. Although the in-person sessions were reported to be essential for discussion of content being learned, offering multimodal learning opportunities provided flexibility and a model for moving the program forward in the future.

CN participants reported that improvements in self-assessed leadership skills after the 4-month leadership course benefited their careers, patients, and teams. The AI framework prompted participants to engage in personal discovery and then create an actionable plan for application and implementation of content learned at each class. Compared with the average turnover cost of $46,100 per direct care nurse, the cost per CN participant was profoundly less, averaging $709 per participant or $8,508 for the program.^28^ The estimated cost of mandating the course for all CNs is $78,699, which is still less than the cost of turnover for two nurses. Investing in frontline leader education and self-development could improve the work environment and influence staff retention. The CNLDWS is one method that can be used as a part of ongoing education. These findings bring a new awareness to the benefits of CN leadership development with fresh consideration for course content and delivery to support the essential role of frontline nurse leaders.
